# The Contribution of the Internet to Reducing Social Isolation in Individuals Aged 50 Years and Older: Quantitative Study of Data From the Survey of Health, Ageing and Retirement in Europe

**DOI:** 10.2196/20466

**Published:** 2022-01-03

**Authors:** Patrícia Silva, Alice Delerue Matos, Roberto Martinez-Pecino

**Affiliations:** 1 Communication and Society Research Centre University of Minho Braga Portugal; 2 Department of Sociology, Institute of Social Sciences University of Minho Braga Portugal; 3 Departamento de Psicología Social, Facultad de Comunicación Universidad Sevilla Seville Spain

**Keywords:** social isolation, internet, 50+ individuals, e-inclusion, SHARE

## Abstract

**Background:**

Social isolation has a negative impact on the quality of life of older people; therefore, studies have focused on identifying its sociodemographic, economic, and health determinants. In view of the growing importance of the internet as a means of communication, it is essential to assess whether internet use interferes with social isolation.

**Objective:**

This study specifically aims to clarify the relationship between internet use and social isolation of individuals aged ≥50 years, for which other surveys present contradictory results.

**Methods:**

We performed logistic regression analysis with social isolation as the dependent variable, internet use as the interest variable, and several other sociodemographic, economic, and health characteristics of the individuals as control variables. The sample size was 67,173 individuals aged 50 years and older from 17 European countries (Portugal, Greece, Italy, Spain, Denmark, Sweden, Austria, Belgium, France, Germany, Switzerland, Luxemburg, Poland, Czech Republic, Slovenia, Estonia, and Croatia) plus Israel, who were interviewed in the Survey of Health, Ageing and Retirement in Europe (SHARE), wave 6.

**Results:**

The results show that countries differ in the level of social isolation and rate of internet use by individuals aged 50 years and older. They also evidence that in most of the countries analyzed, social isolation of internet users was lower compared to that of nonusers after controlling for a set of sociodemographic, economic, and health characteristics of the individuals that have been previously described in the literature as determinants of social isolation. Indeed, on average, although 31.4% of individuals in the nonuser group experienced high social isolation, only 12.9% of individuals who used the internet experienced this condition.

**Conclusions:**

Internet users show lower social isolation. This result underlines the importance of promoting e-inclusion in Europe as a way to counter social isolation of individuals aged 50 years and older.

## Introduction

Social isolation has been defined as the objective situation of individuals with small social networks and reduced frequency of contact who do not take part in social activities [1-4]. According to the literature, social isolation is associated with an increased risk of mortality [2,5-7]. In terms of health, it has been associated with a greater risk of developing chronic diseases [8] and cardiovascular disease [3,7], as well as increased risk of physical inactivity, tobacco consumption, and various other risk behaviors [3,9]. Furthermore, in old age, social isolation is associated with increased feelings of loneliness [3].

According to the literature, social isolation tends to increase as individuals age [3,10,11]. Indeed, some events that are more frequent at an older age, such as retirement, the development or worsening of health and mobility limitations, or a change in residence of the individuals with whom they socialize [2,12-16], tend to affect older people’s ability to maintain their social networks and may also cause cognitive decline [17] and increase social isolation [2].

Social isolation is determined by a set of sociodemographic, economic, and health characteristics of individuals [3,10,13,18,19]. In addition to these determinants, the role of the internet in reducing social isolation is increasingly being discussed [20] because, as an important means of communication, this technology can facilitate interpersonal contact at a stage of life when social networks tend to be restructured [12,21-24]. There are often obstacles to the use of the internet by people of advanced age [25-31]. However, internet use by older adults has been associated with feelings of well-being and social support [32-35], as well as with improved quality of life [36]. Nevertheless, the relationship between internet use and social isolation is unclear, and there is open debate in the literature. On the one hand, some studies conclude that internet use is associated with a decrease in social isolation [11,20,37,38]. In this regard, they claim that the internet has been conducive to successful interactions [39-41] and contributes to maintaining social ties [41-44] and increasing the frequency of contact [45], as well as optimizing the effect of social networks on the quality of life of older people [36]. On the other hand, other studies conclude that using this technology does not reduce social isolation [46]. Furthermore, in some specific cases, its use is actually associated with a greater risk of social isolation. In this regard, problematic and addictive uses of the internet may be related to social isolation [47]. Similarly, some studies support the “time displacement” thesis, which states that the longer individuals surf the internet, the less time they interact with family and friends, as the time spent on one activity cannot be spent on another [48].

The inconsistent results in the literature underscore the need for more research into the relationship between internet use and social isolation [20,49], in which this concept is clearly defined [50] and which draws on large samples [38,51]. This study specifically aims to contribute to this goal by focusing on the relationship between internet use and social isolation after controlling for sociodemographic, economic, and health variables and selecting a target population of individuals aged 50 years or older residing in 17 European countries and Israel.

## Methods

### Data and Sample

This study focuses on 67,173 individuals aged 50 years and older who were interviewed in the Survey of Health, Ageing and Retirement in Europe (SHARE) Project (wave 6) in Austria (n=3358), Germany (n=4347), Sweden (n=3881), Spain (n=5560), Italy (n=5211), France (n=3870), Denmark (n=3661), Greece (n=4814), Switzerland (n=2772), Belgium (n=5700), Israel (n=2013), Czech Republic (n=4793), Poland (n=1802), Luxembourg (n=1543), Portugal (n=1662), Slovenia (n=4186), Estonia (n=5557), and Croatia (n=2443). Details of the SHARE study in Europe have been described elsewhere [52]. Briefly, in wave 6 (2015), a survey of a representative sample of the noninstitutionalized population aged 50 years or older was conducted. Interviews were face-to-face and took place in the household. Trained interviewers conducted the interviews using a computer-assisted personal interviewing program.

The SHARE project, coordinated internationally by the Max Planck Institute for Social Law and Social Policy (Germany), has been approved by the Ethics Council of the Max Planck Society for the Advancement of Science.

### Measures

#### Dependent Variable: Social Isolation

As in other research [3,4,53], social isolation was computed using a 5-item index. Individuals scored 1 point if they did not live with a partner; 1 point if they did not belong to any organizations, clubs, or religious groups; and 1 point for having less than monthly contact with friends, family, or children. Scores on the index ranged from 0 to 5, with higher scores indicating a greater degree of isolation. Adopting the criteria of previous studies [2,54-56], we dichotomized the social isolation index to distinguish between low (score <2) and high (score ≥2) levels of social isolation.

Low social isolation was coded as 0, and high social isolation was coded as 1.

#### Independent Variable: Internet Use

This is a dichotomous variable relating to use of the internet at least once in the last 7 days to send and receive emails, to search for information, to shop, or for any other purpose. This variable distinguishes individuals who use the internet (1) from individuals who do not use it (0).

#### Covariables: Sociodemographic and Economic Variables

The following covariables were considered in this research:

- Age; gender: female (1) and male (0); years of schooling; self-perception of financial stress: “great difficulty” or “some difficulty” in coping with monthly expenses (0), “easy” or “very easy” to cover monthly expenses (1).
- Geographical distance from social network: scores ranged from ”in the same household” (1) to “more than 500 km away” (8).
- Loneliness: assessed through a short version of the Revised UCLA Loneliness Scale (R-UCLA) [57]. The scale includes 3 questions: “How much of the time do you feel you lack companionship?” “How much of the time do you feel left out?” and “How much of the time do you feel isolated from others?” The answer options range from 1 (hardly ever) to 3 (often). The 3 items form a scale that ranges from 3 to 9 points, in which high values represent higher levels of loneliness.

We also considered health variables:

- Depressive symptoms, evaluated by the EURO-D scale [58]. The EURO-D scale ranges from 0 to 12 points, which refer to the presence or absence of 12 symptoms of depression, such as suicidal thoughts [58]; as in previous studies [59], we distinguished between more symptoms (1) and individuals with lower scores (0). A score of 4 or more symptoms is considered as a cutoff point to identify major depression [60].
- Limitations in activities of daily living [61,62], which refer to the presence-absence of difficulties performing on one’s own any of 6 daily living activities, such as bathing, dressing, and toileting; we distinguished between individuals who reported 1 or more limitations (1) and individuals who reported no limitations (0).
- Physical inactivity: Two items were used to assess the level of physical activity. The first item assessed vigorous physical activity (“We would like to know about the type and amount of physical activity you do in your daily life. How often do you engage in vigorous physical activity, such as sports, heavy housework, or a job that involves physical labor?”). The second item assessed moderate physical activity (“How often do you engage in activities that require a low or moderate level of energy such as gardening, cleaning the car, or doing a walk?”). Participants answered both items by using a 4-point rating scale (1, more than once a week; 2, once a week; 3, 1 to 3 times a month; 4, hardly ever or never). Participants who did not perform any of these activities (never engaged in vigorous or moderate physical activity) were classified as “physically inactive” (1), and individuals who performed at least one vigorous or moderate physical activity were classified as “physically active” (0).

### Statistical Analysis

Given that the SHARE project includes national samples from different countries and the sample design is not uniform among them, calibrated individual weights were used in the descriptive statistical analysis (for further details, see [63,64]). To compare sociodemographic, economic, and health characteristics between individuals who experience high levels of social isolation and individuals with low levels of social isolation, the chi-square test and Student *t* test were performed. We used the chi-square test to assess the interdependence between two nominal variables (eg, differences between gender according to their degree of social isolation). The sample means were also compared using Student *t* tests for independent samples (eg, differences in mean age according to their degree of social isolation). Statistical test results with *P*<.05 were considered to be significant. The results from these tests were complemented with effect size measures (Cohen *d*/*φ*). The interpretation of the results was based on Cohen [65].

To analyze the relationship between internet use and social isolation, logistic regressions were conducted by country using the Enter method (in which all the dependent variables are inserted in the statistical model simultaneously). The logistic regression is a mathematical model that give odds ratios (ORs) that are adjusted for other covariates (including confounders) [66]. Thus, 18 separate analyses were performed (1 for each country). Weights were not used in the logistic regressions. These analyses were performed using SPSS software (version 25; IBM Corp).

## Results

Eastern and Southern Europe are the opposite of Northern and Central Europe in terms of social isolation (Table 1). In fact, the percentage of individuals aged 50 years and older who are socially isolated is higher in Eastern and Southern Europe (with the exception of Portugal) compared to the two other European regions. An analysis by country confirmed that the highest weighted percentage of people who experience a high level of social isolation is found in Poland (n=463; 36.8%), and the lowest is found in Denmark (n=321; 11.8%).

With regard to internet use, the countries of Northern and Central Europe also stand out in terms of recording the highest percentages of users, with Denmark once again showing the highest rate of use of technology (n=3053; 81.9%). In contrast, the countries of Eastern and Southern Europe have the lowest rates of internet use, with Croatia standing out as a country where fewer individuals use the internet (n=715; 27.2%).

**Figure 1:**
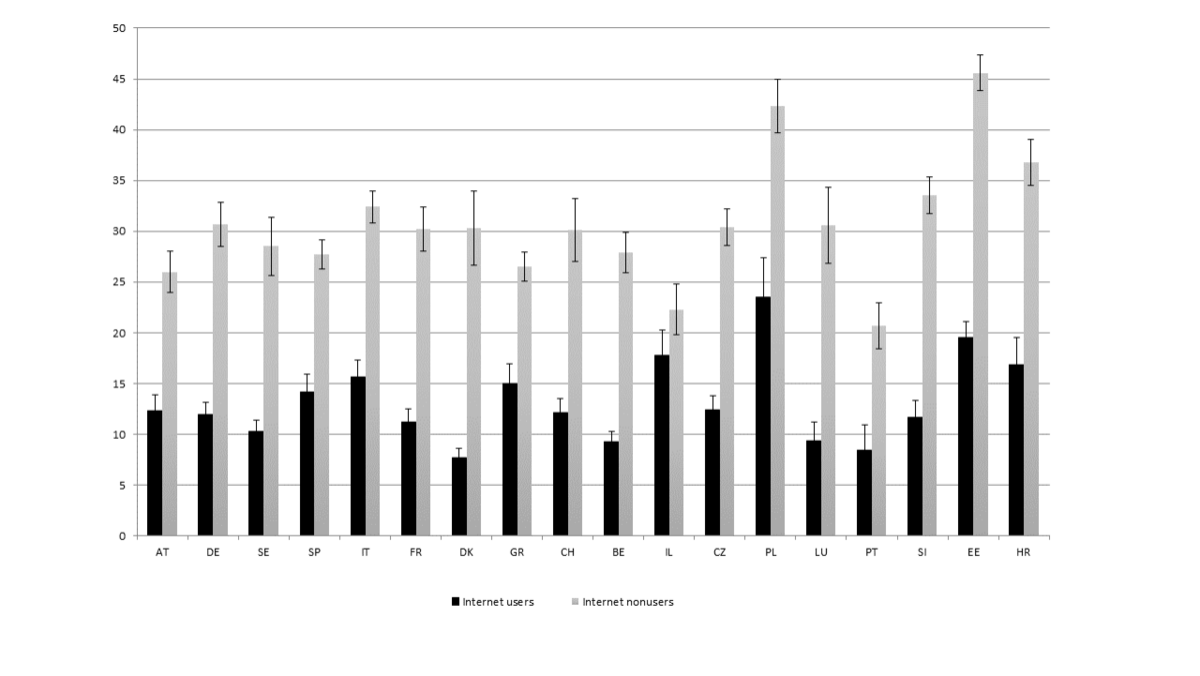
Percentages of users and non–internet users in the high isolation group by country.

In all the countries analyzed, high levels of social isolation are clearly more common among individuals who do not use the internet as compared to those using this technology (Figure 1). Indeed, while on average, in the group of internet users, only 12.9% experience high levels of social isolation, in the group of nonusers, 31.4% are in this situation.

**Table 1:** Individuals in the high isolation group (n=11,614) and internet users (n=32,399) by country (N unweighted). Source: Survey of Health, Ageing and Retirement in Europe wave 6, version 7.0.0; weighted data.

Country	High social isolation group, n (%)	Internet users, n (%)
Poland	463 (36.8)	447 (28.1)
Estonia	1408 (33.0)	2490 (47.6)
Croatia	486 (31.2)	715 (27.2)
Italy	914 (26.6)	1698 (33.6)
Slovenia	726 (24.5)	1564 (40.9)
Greece	1010 (23.3)	1265 (27.9)
Spain	946 (22.6)	1655 (37.5)
Czech Republic	1028 (21.2)	2268 (50.9)
Israel	307 (20.0)	949 (50.0)
Germany	552 (19.8)	2592 (57.6)
Austria	602 (18.8)	1606 (52.6)
France	739 (18.8)	2178 (59.8)
Luxembourg	184 (18.0)	955 (59.6)
Switzerland	395 (17.2)	1912 (71.4)
Portugal	231 (16.9)	451 (30.8)
Belgium	891 (15.8)	3684 (65.1)
Sweden	411 (14.1)	2917 (78.6)
Denmark	321 (11.8)	3053 (81.9)

In Table 2, we can observe the sociodemographic, economic, and health characteristics of the individuals who participated in this study, according to their degree of social isolation. The group of individuals who experience high levels of social isolation includes a majority of older female adults, who have on average fewer years of schooling in relation to their counterparts experiencing low levels of social isolation. Similarly, a higher percentage of the more socially isolated individuals reported having a negative financial situation.

With regard to social networks, the more isolated individuals are more geographically distant from members of their network than their less isolated counterparts. Likewise, more socially isolated individuals also experience greater feelings of loneliness.

Finally, with regard to mental and physical health, individuals experiencing high levels of social isolation also report more depressive symptoms and physical inactivity than their less isolated peers.

In Table 3, we can observe a negative association between internet use and social isolation, after controlling for a set of sociodemographic, economic, and health characteristics of the individuals, described in the literature as determinants of social isolation. Indeed, in most of the countries analyzed, internet use relates to a decreased likelihood of high levels of social isolation.

Denmark is the country where internet users are less likely to experience high levels of social isolation compared to nonusers. In this country, internet users are 72.2% less likely (OR 0.278, 95% CI 0.196-0.396) to be in a situation of high social isolation. Similarly, in France, Sweden, Luxembourg, Germany, Czech Republic, Estonia, Switzerland, Austria, Italy, Slovenia, and Spain, internet users are less likely to experience high levels of social isolation than nonusers. In Portugal, Poland, Greece, and Croatia, internet users were not less likely to experience high social isolation. Outside the European context, more specifically in Israel, internet users were 42.7% less likely (OR 0.573, 95% CI 0.383 to 0.856) to experience high levels of social isolation.

**Table 2:** Descriptive statistics of the variables studied according to degree of social isolation (N=67,173). Source: Survey of Health, Ageing and Retirement in Europe wave 6, version 7.0.0; weighted data.

Variable				High social isolation (n=11,614)		Low social isolation (n=55,559)		*χ*^2^/t (*df*)		*P* value		Effect size (Cohen *d/φ*)
**Sociodemographic and economic characteristics**												
	Age (years), mean (SD)			70.05 (11.95)		65.07 (10.26)		48.781 (1)		<.001		0.498 (small)
	**Sex, n (%)**							1457.933 (1)		<.001		0.147 (small)
		Female	8359 (67.6)		29,243 (50.3)							
		Male	3255 (32.4)		26,316 (49.7)							
	Schooling (years), mean (SD)			9.51 (4.26)		11.17 (4.51)		–32.922 (1)		<.001		0.344 (small)
	**Financial situation, n (%)**							976.178 (1)		<.001		0.122 (small)
		Positive	5309 (48.1)		34,471 (64.5)							
		Negative	5799 (51.9)		19,669 (35.5)							
	Geographical distance from social network (km), mean (SD)			3.89 (1.57)		3.06 (1.61)		58.917 (1)		<.001		0.652 (medium)
	Loneliness, mean (SD)			4.66 (1.78)		3.78 (1.27)		52.414 (1)		<.001		0.540 (medium)
**Health**												
	**Exhibits depressive symptoms, n (%)**							1327.652 (1)		<.001		0.144 (small)
		Yes	4697 (43.0)		12,751 (25.9)							
		No	6713 (57.0)		39,556 (74.1)							
	**Limitations in activities of daily living, n (%)**							662.02 (1)		<.001		0.099 (trivial)
		≥1	2253 (18.9)		5977 (11.0)							
		None	9358 (81.1)		49,440 (89.0)							
	**Physical activity, n (%)**							925.115 (1)		<.001		0.117 (small)
		Inactive	2434 (23.5)		5929 (12.2)							
		Active	9178 (76.5)		49,473 (87.8)							

**Table 3:** Statistics related to the importance of the internet as a determinant of a high level of isolation in individuals aged ≥50 years. Data source: Survey of Health, Ageing and Retirement in Europe wave 6, version 7.0.0 (unweighted).

Country^a^	B^b^	aOR^c^ (95% CI)	*P* value
Austria	–0.616	0.540 (0.419-0.697)	<.001
Germany	–0.797	0.451 (0.352-0.576)	<.001
Sweden	–0.842	0.431 (0.316-0.588)	<.001
Spain	–0.328	0.720 (0.558-0.931)	.01
Italy	–0.554	0.574 (0.415-0.795)	.001
France	–0.909	0.403 (0.312-0.520)	<.001
Denmark	–1.279	0.278 (0.196-0.396)	<.001
Greece	–0.062	0.940 (0.717-1.232)	.65
Switzerland	–0.675	0.509 (0.376-0.690)	<.001
Belgium	–0.808	0.448 (0.356-0.557)	<.001
Israel	–0.557	0.573 (0.383-0.856)	.007
Czech Republic	–0.795	0.452 (0.367-0.557)	<.001
Poland	–0.166	0.847 (0.521-1.377)	.50
Luxembourg	–0.802	0.448 (0.275-0.732)	.001
Portugal	0.351	1.421 (0.862-2.342)	.168
Slovenia	–0.500	0.606 (0.430-0.856)	.004
Estonia	–0.788	0.455 (0.371-0.557)	<.001
Croatia	–0.300	0.741 (0.529-1.038)	.08

^a^Unweighted n values: Austria, 2523; Germany, 3853; Sweden, 3205; Spain, 4079; Italy, 3297; France, 2904; Denmark, 3110; Greece, 4128; Switzerland, 2329; Belgium, 3999; Israel, 1242; Czech Republic, 3941; Poland, 1161; Luxembourg, 1394; Portugal, 1284; Slovenia, 2523; Estonia, 4154; Croatia, 2274.

^b^B: Standardized Coefficients

^c^aOR: adjusted odds ratio from the logistic regression with adjustment for age, gender, years of schooling, self-perception of financial stress, limitations to activities of daily life, EURO-D score, physical inactivity, geographical distance from social network, and loneliness.

## Discussion

### Principal Findings

The aim of this study was to analyze the relationship between internet use and social isolation of individuals aged ≥50 years. The results evidence that in most of the European countries analyzed, as well as in Israel, use of the internet by adults aged ≥50 years is related to a decreased likelihood of high levels of social isolation. Thus, this study contributes to the open debate in literature [37,50] for which other surveys present contradictory results. This could be related to the results of other studies [11,20,67] that suggest that the internet may facilitate communication and therefore enable individuals to maintain important contacts [36,39,68]. The internet may therefore contribute to less social isolation, even in less favorable contexts [12], such as when older people live at a greater geographical distance from elements of their social networks following retirement, migration, or a change in residence for other reasons [2,12-15]. However, the impact of ICT cannot be explained solely in terms of the fact that it creates opportunities for communication. The literature reveals that it counters social isolation by enabling people to obtain social support and motivating individuals to participate more in activities that interest them, because it contributes to self-confidence [37] and facilitates access to services [69].

In this study, it was also possible to verify that in the European context, countries in Eastern and Southern Europe had the highest percentages of individuals experiencing high levels of social isolation. This result is consistent with results in other studies comparing the northern European countries with southern European countries, which indicate the existence of greater social isolation in the southern countries [70]. In line with other research [3,10,11], this study concludes that high levels of social isolation are more common in older female individuals [71]. This finding contradicts the results of other research that identifies men as being more isolated than women [13,18]. The results also reveal that the most isolated individuals also have more financial difficulties, as noted in other research [3,13,18], and they experience higher levels of loneliness, as also noted in other research [19]. In this study, individuals in situations of high social isolation also reported being more geographically distant from their social network. The impact of increased geographical distance on establishing social interactions at an older age was previously underlined by other surveys showing that one of the main reasons for losing elements of adult social networks is a change in residence of these individuals [72]. In terms of health, people experiencing high levels of social isolation also report being frailer, both physically and mentally [3,18].

However, as previously mentioned, the main contribution of the study is that after controlling for the impact of these variables, internet use is associated with lower risk of social isolation.

### Limitations

This study has several limitations. The main limitation is that a single item was used to measure overall internet use. Nevertheless, a yes/no response to whether someone regularly uses the internet has frequently been used to assess internet use by older people [25,45,51,67]. In the same vein, this measure was used in the SHARE project. Nonetheless, considering that the impact of the internet in the social sphere depends on the type of activities conducted on the web [73-75], it is important for future studies to consider the impact that different uses of the internet may have on social isolation. Furthermore, future research should consider longitudinal analyses to explore causality.

### Conclusion

The results of this research contribute to the scientific debate about the relationship between internet use and social isolation, showing that even after controlling for the main determinants of social isolation, the use of the internet is related to lower levels of isolation in several countries.

These results indicate the importance of developing public policies in Europe aimed at increasing rates of internet use as a way to ensure e-inclusion and prevent social isolation at an older age.

## Abbreviations

OR: odds ratio
R-UCLA: Revised UCLA Loneliness Scale
SHARE: Survey of Health, Ageing and Retirement in Europe
